# Editorial: Precision trait mapping and molecular breeding in high-impact crop plants

**DOI:** 10.3389/fgene.2025.1736266

**Published:** 2025-11-17

**Authors:** Dinesh Kumar Saini, Mamta Gupta, Alla Singh, Mehak Sethi, Veena Devi, Reyazul Rouf Mir

**Affiliations:** 1 Department of Plant and Soil Science, Texas Tech University, Lubbock, TX, United States; 2 ICAR-Indian Institute of Maize Research, Ludhiana, India; 3 Faculty of Agriculture, Sher-e-Kashmir University of Agricultural Sciences and Technology of Kashmir, Srinagar, India

**Keywords:** molecular breeding, trait mapping, translational breeding, candidate gene, genetic gain

## Introduction

Modern crop improvement is going through a major transformation. The convergence of high-resolution genotyping, phenomics, and bioinformatics now enables researchers to uncover the genetic architecture of complex traits with unprecedented precision. This integration has reshaped breeding pipelines from single-trait selection toward systems that simultaneously target yield, resilience, and nutritional quality. The Research Topic “*Precision Trait Mapping and Molecular Breeding in High-Impact Crop Plants*” in *Frontiers in Genetics* features six studies that tackle key breeding challenges across cereals, legumes, and oilseeds. Collectively, these papers illustrate how cutting-edge molecular tools, genomics, and translational breeding strategies can be deployed to dissect traits, harness genetic diversity, and design future-ready crop ideotypes capable of addressing both productivity and nutritional demands.

## Precision breeding for nutritionally enriched wheat

Improving the nutritional quality of staple cereals is a global priority. Two studies in this Research Topic by Kapoor et al. and Kumar et al. focus on dietary fibre biofortification in wheat. Kapoor et al. analyzed 478 genotypes including cultivars, tetraploids, and wild relatives, and found large variation in β-glucan and arabinoxylan content. Wild *Aegilops* species, especially *A. peregrina* and *A. kotschyi*, showed higher fibre and protein levels, highlighting their value for improving wheat nutrition. Building on this, Kumar et al. used precision breeding to introduce high β-glucan alleles from *A. kotschyi* into hexaploid wheat. Using marker-assisted selection and cytogenetic validation, they developed BC_2_F_2:3_ lines with up to 1.76% β-glucan, along with yellow rust resistance and good agronomic traits. Together, these studies demonstrate a complete pathway from genetic diversity exploration to precision introgression, showing how molecular tools and classical breeding can together enhance the nutritional quality and agronomic performance of wheat.

## Dissecting complex trait architecture in sorghum and maize

Crop improvement increasingly depends on high-resolution trait mapping to speed up the discovery of genes controlling yield, stress tolerance, and quality. Two studies in this Research Topic highlight this shift toward precision genomics in complex trait analysis. In sweet sorghum, Umar et al. conducted a genome-wide association study (GWAS) on 183 accessions using 14,819 high-quality SNPs and identified 21 significant QTNs linked to agronomic and sugar-related traits, explaining 5%–14% of variation. Key candidate genes were linked to flowering, ethylene response, and biomass accumulation, revealing how carbon partitioning and growth timing shape bioenergy potential. In maize, Kaur et al. addressed resistance to the stem borer *Chilo partellus*, a major tropical pest, using an F_6_ recombinant inbred population derived from cultivated maize (LM13) and teosinte (*Zea mays* ssp. *parviglumis*). Using SSR and SNP markers, they identified four QTLs (*qLIR_4.1*, *qLIR_9.1*, *qDH_1.1*, and *qDH_2.1*) associated with resistance traits such as leaf injury rating and dead-heart percentage. This represents one of the first reports of *C. partellus* resistance QTLs in Asia and highlights teosinte as a valuable source of pest resistance alleles. Together, these studies show how population genomics and wild introgression can enhance resilience and resource-use efficiency, key foundations for sustainable crop improvement.

## Meta-analysis and translational genomics for yield improvement in legumes

While cereals dominate global caloric supply, legumes remain essential for protein and micronutrient security. Du et al. contributed a landmark synthesis through the comprehensive meta-analysis of yield and yield-related quantitative trait loci (QTLs) in mungbean (*Vigna radiata*). By consolidating 660 QTLs reported over 2 decades, the study refined them into 72 meta-QTLs (MQTLs) with sixfold narrower confidence intervals and validated 20 through independent GWAS studies. Beyond data integration, the study also examined colinearity between mungbean and common bean genomes, revealing 22 orthologous MQTLs associated with conserved yield determinants such as seed size, tiller number, and plant height. The identification of breeder MQTLs, regions with clusters of favorable alleles supported by literature and validation, represents a key step toward practical use. This approach shows how meta-genomics and comparative analysis can turn scattered QTL data into usable targets for marker-assisted and genomic selection in legumes.

## Harnessing cytoplasmic engineering for hybrid seed innovation in oilseeds

Hybrid seed technology is one of the most effective ways to exploit heterosis, but its success depends on stable male sterility and fertility restoration systems. Addressing this challenge, Wang et al. developed a new breeding approach for *Brassica napus* (rapeseed) by creating Ogura cytoplasmic male sterility (CMS) restorer lines without relying on external restorer sources. By crossing a doubled haploid induction line with Ogura CMS plants, they unexpectedly obtained fertile offspring with 97.7% mitochondrial genome similarity but with key nuclear differences that restored fertility. Bulked segregant analysis (BSA) mapped restorer gene candidates to three regions, A09 (10.99–17.20 Mb), C03 (5.07–5.34 Mb), and C09 (18.78–36.60 Mb), showing that restorer alleles can be induced naturally through genomic recombination. This breakthrough removes reliance on radish derived sources and provides a useful model for hybrid development in *Brassica*. The study shows how combining cytoplasmic engineering with genomic mapping can strengthen hybrid seed production and support both yield gains and genetic diversification in oilseed breeding.

## Conclusion

Across these six studies, a common message emerges: precision trait mapping connects molecular insights with real breeding outcomes. From wild gene pools and cytogenetic introgression to GWAS, QTL analysis, and restorer systems, these works show how plant breeding is becoming a data-driven and integrated science. Looking ahead, combining molecular tools with advanced phenotyping, multi-omics data, and machine learning will help breeders design purposeful, high-performing crop varieties. Such predictive frameworks can deliver cultivars that are more nutritious, resilient, and efficient, advancing both productivity and sustainability. This Research Topic highlights major progress in trait mapping and molecular breeding while charting the path toward truly precision-driven agriculture.

